# Is There an Epidemic of Scabies in the State?

**Published:** 1906-04

**Authors:** Bernard Wolff

**Affiliations:** Atlanta, Ga.


### IS THERE AN EPIDEMIC OF SCABIES IN THE STATE?*

BY BERNARD WOLFF, M.D.,

ATLANTA, GA.

For several months past I have been impressed with the unusual frequency with which cases of scabies have occurred in my practice. The number was so abnormally large that I was forced to the conclusion that the disease was either endemic or epidemic. For the former, owing to the contagious nature of the affection and the amount of travel to and from this city, there appeared little justification. In order to test the correctness of the epidemic theory, I wrote to a number of physicians located at widely separated portions of the State with the request that they inform me whether or not they had observed an unusual number or a very considerable number of cases of itch in their practice during recent months. This method of inquiry was adopted to gain an idea of the extent of distribution of the disease and its relative severity. The response was courteously prompt and covered a large part of the State geographically. The answers disclosed an almost unanimous testimony to the wide prevalence of the disease. In almost every locality subjected to this investigation a large number of people were or had been affected with the itch. It was by no means limited to the lower classes, including colored people, but had permeated every stratum of society. Dr. S. A. Brown, of Dalton, Whitfield county, informs me that the disease prevails to a considerable extent in his county. He attributes it to the returned soldiers from the Spanish-American War. Dr. Bush, of Pelham, Mitchell county, the opposite end of the State from Dr. Brown, has seen between forty and fifty cases from October 1 to January 1. These cases were not among the poor whites and negroes, but among some of the best people in his town and county. He speaks in the highest praise in the

*Read by title at the Covington meeting of the Medical Societies of DeKalb, Rockdale and Newton counties, March 20.

treatment of the disease of a combination of alcohol, castor oil, sulphur and oil of bergamot. Dr. Corry, of Barnesville, Pike county, has also seen forty or fifty cases, and states that it is rife in his county, and that it is epidemic among the pupils of Gordon Institute. Dr. L. G. Hardman, of Commerce, has seen a large number of cases, and asserts that it is distributed all over his county. Dr. Stewart Roberts, of Emory College, Oxford, saw eight cases during the session of 1904-05, but has seen only one case since September, 1905. He is inclined to think that a factor in the spread of the disease throughout the State was the great amount of travel incident to the St. Louis Exposition.

Dr. Jeff. Davis, of Toccoa, says there has been in his locality a large number of itch cases, and that they "keep a comin'." Dr. G. L. Alexander, of Forsyth, has seen about a dozen cases of scabies in the last six months, and has been surprised to find it existing in the best families in his clientele. He accounts for the trouble being unusually common on the score of faulty diagnosis and insufficient precautions being taken against its spread. He has practiced in Forsyth since 1893 and has seen very few itch cases until the past six months. Dr. W. D. Travis, of Covington, thinks that there have been something like fifty cases of scabies in his county during the past fall and winter. Dr. Henry E. Thornton, of Hartwell, Hart county, seems to be somewhat in the storm centre. He has treated since January 1 twenty-one cases and Dr. Geo. S. Clark, of the same town, states that he has treated about one hundred cases of itch within the last twelve months. Three of Dr. Thornton's patients were traveling salesmen, a fact that may be suggestive of another vehicle of dissemination. Dr. Eugene R. Corson, of Savannah, has had recently more cases of itch than he can remember to have had for some time. He has had nine cases, several of the patients being from Blackshear, Ga., and its vicinage. Dr. L. J. Belt, of Millen, was more moderately circumstanced. He has had one case and heard of one other, these being the only ones that have come within his observation for a number of years. He saw many cases ten years ago. Dr. Sanchez, of Barwick, knows of between twenty-five and fifty families in his locality affected with itch. Dr. J L. Walker, of Waycross, has had about thirty cases. Dr. P. L. Hilsman, of Albany, has had an unusual number of cases of scabies in the last few months. One if his patients contracted the disease from a room-mate in Atlanta. Dr. Hilsman saw a great deal of itch during the Civil War. It not only overran the army, but was brought home by returning soldiers, so that scarcely any one escaped. He has seen more or less of the disease since the war with Spain. He thinks that the disease was spread at that time, but it has been more general in his locality within the past few months than during and immediately after this war. Dr. Hilsman is also kind enough to furnish me with the prescription he is in the habit of using. It is a combination of ammoniate of mercury, balsam of Peru and the compound sulphur ointment of Hebra—certainly a formidable array to bring to bear against the sinning acarus. Dr. J. K. Burns, of Clarkesville, Habersham county, has treated about forty or fifty cases during the last twelve months. It has been scattered about in his county since the Spanish-American war. He believes that the soldiers brought it thither from Cuba. Dr. J. R. Burdett, of Tennille, has not seen any scabies recently. About two or three years ago it was very thoroughly distributed in his locality.

Dr. Henry R. Slack, of LaGrange, has not seen any cases of the disease for a year. He believes his locality to be free of it. Dr. A. K. Bell, of Madison, Morgan county, has not seen a single case for two years, and has heard of none. I have had one case from Madison and another from Morgan county, so that I am quite confident that scabies exists there, even though it has appeared to have escaped Dr. Bell's notice. Dr. C. M. Curtis, of the neighboring village of College Park, tells me that he has twenty-five cases among the students of the Georgia Military Academy. I also am reliably informed of the excessive prevalence of the disease at Athens, Alpharetta, Royston, Dahlonega, and other places about the State not covered by a specific inquiry.

Without tedious multiplication of instances, the conclusion appears to be justified that scabies has permeated the State in every direction and merits the epithet of epidemic. There are localities here and there where the disease has apparently touched lightly and passed on, or has avoided altogether, but it is manifest from the testimony offered by the replies received from my letters that these localities are comparatively few, when measured against the infected areas. The disease has affected all classes of society, and has well illustrated the interdependence of man as a social being.

If the suggestions offered by some of my correspondents concerning the origin of the disease, or of the present widespread dissemination of it, are to be regarded as established fact rather than opinion, it was introduced by soldiers returning to their homes after having been mustered out subsequently to the war with Spain. The disease, acquired in Cuba, where it is perennial, was thus conveyed broadcast throughout the country towns and cities of the South and elsewhere. Transplanted in this way, it spread its borders and steadily increased. Each case became a focus of distribution in an ever-widening circle. This theory, while it lacks definite evidence, has much that commends it as being correct, and it is very likely, indeed, that the present epidemic found its source in the dispersion of the volunteer troops after the Spanish-American War of 1898. A score of years ago scabies was one of the uncommon diseases of the skin in the United States, but it now, according to the statistics of the American Dermatological Association, constitutes about eight per cent. of all cases of skin disease. After the general diffusion of the disease which followed the close of the war between the States, as is stated by Dr. Hilsman, very few escaped. It gradually diminished in its ravages until it became comparatively infrequent. In later years the enormous influx of emigrants from the Continent of Europe brought numberless cases of scabies to this country, and the disease was spread widely throughout the North and West. The South, with its exceedingly small percentage of alien population, was not invaded to any extent, and it remained for another war to repeat what promises to be the same condition as that which followed the Civil War.

*Spread and Distribution.*—There are several factors determining the spread of the disease. The initial velocity of the invasion in force offered by the returned soldiers carried it well on its way of progress, and established a great many foci or depots of distribution which, after a time, widened, coalesced and covered wide geographical areas.

The previous infrequency of the disease in the State had not furnished the diagnostic experience to any except the older physicians which insured a ready identification of the eruption. As a consequence, errors of diagnosis were not infrequent, and the result in the case of a contagious disease can better be imagined than expressed. A further factor in the spread of the disease was the lack, in many instances, even when the nature of the eruption was well known, of proper precautions to prevent its spread. The thorough disinfection of bedding and wearing apparel not being insisted upon, scabies first became a personal affection, later familial, and finally communal and general. A fortunate circumstance, in this connection, is that scabies requires prolonged contact for transmission. Casual, brief contact, such as that offered by shaking hands, is hardly sufficient. Were it not for this fact, the unwarranted negligence of patients and the remissness of the profession in not sufficiently warning them against the danger of contagion, the epidemic would, without doubt, have attained much greater dimensions than at present.

*Clinical Variations.*—As the eruption of scabies is due to the irritation offered by the presence of the female acarus in the epidermis, it is but natural to presume that the lesions produced will vary in form and number in proportion to the sensitiveness of the skin and the amount of irritation aroused, with the subsequent injury inflicted by the patient upon himself by scratching. With the exception of the cuniculus, there is nothing distinctive about the lesion of scabies, but it is readily recognized in a typical instance by the multiformity of the lesions and their peculiar distribution. The acarus prefers the regions of the body where the skin is thin, as it may in such regions gain ready access and, tunneling its way through the epidermis, form its characteristic burrow or cuniculus. This latter appearance, when seen, establishes the diagnosis beyond quibble, but, in my experience at least, it is not such an easy task always to find it. The burrow is often crusted over and lost in the general upheaval of the skin caused by violent scratching. The regions where the acarus is most likely to be found are the opposing surfaces of the shafts of the fingers, the ulnar border of the hands, axillary spaces, the skin of the penis. It may be recognized in a cleanly person as a whitish sinuous or straight line, a little reddened at one end. If a needle is inserted at this reddened extremity the acarus may be dislodged. It appears to the eye as a whitish speck, barely visible and considerably smaller than the chigoe or common redbug. A deliberate and careful search should always be made for the acarus. Once it has been found and identified microscopically the exact character of the disease, in spite of its disguises, is immediately apparent, and appropriate treatment soon effects a cure. These disguises constitute the clinical variations from the typical manifestations of the disease, and are of great diagnostic importance. One must rid himself of the notion that itch is always a vesiculo-pustular dermatitis affecting certain localities. This is the correct picture of neglected cases, in which the disease has been allowed to run its course unchecked, in a person of uncleanly habits or a child. The eruption is often greatly modified by the habits of the host of the parasite, and varies in its morphological appearances within very wide limits. The lesions may consist of papules, pustules, vesicles, together with ecthymatous ulcers, folliculitis and cutaneous abscesses or scantily distributed pin-head-sized itchy papules, not especially conspicuous upon the localities of predilection. These marked differences were often noted in the recent series of fifty-nine cases seen by me. For instance, a gentleman, among whose children scabies had appeared, presented a few small, red, itchy papules upon the abdomen, axillary borders and sides of the thorax. There was also considerable general pruritus. The patient promptly got well under sulphur inunctions. Without the assistance of collateral evidence furnished by the indubitable presence of itch in his household, it is likely that the nature of the disease would not have been suspected. In antithesis to this, was a case in which the lesions were exceedingly abundant and of the classical type. The penis was the seat of crusted lesions, eight or ten in number, upon the glans and sheath; the crusts being removed showed shallow ulceration, and yielded rather a marked resemblance to the large pustular syphilide. The ready response to sulphur treatment proved the nature of the eruption, and dispelled the suspicion of syphilis. As a rule the lesions, as to appearance and distribution, have been fairly true to type. Even in cases of sparse eruption, the regions preferred by the acarus have been as a rule involved. A young girl had not more than a dozen lesions upon the general surface; the most peculiar were two large vesicles, almost bullous in size, upon the ulnar border of one hand and the thenar eminence of the other. There were, in addition, several scaling, itchy papules about the mammary areola.

Two young boys presented, as the sole lesion at the time when they were seen, a large dusky-red papule on the glans penis. The diagnosis was made by the existence in the family of other cases of itch.

The cuniculi were easy to find in a number of cases, but in a larger number defied detection, being obliterated by suppuration or crusting, or destroyed by the fingers in scratching.

If scabies is actually epidemic and on the increase, as there seems to be no good reason to doubt, how may it be suppressed? Suppression of the disease may be accomplished in two ways, by early diagnosis and by prompt and thorough disinfection.

The fact of the great prevalence of the disease should prepare the professional mind, and arouse among the physicians of this State and elsewhere an alert watchfulness for the appearance and appearances of the disease. Should the diagnosis be in doubt, any suspicious eruption, especially upon the regions of predilection and characterized by intense nocturnal itching, had better be regarded as scabies and treated as such. This method, while not very scientific, is at least safe, does no harm and, in fact, acts as a therapeutic test.

Proper disinfection includes the destruction of the parasites upon the patient's body and upon his clothing, bedding and the various articles of domestic furniture likely to harbor the itch mite. So far as the first consideration—the treatment of scabies — is concerned, the large list of parasiticides offers a choice of remedies. Among those in general use, and of approved value, are sulphur, beta naphthol, balsam of Peru, storax and mercury. All are effective, and may be used according to personal preference. The stronger preparations should be reserved for use in adults, as the tender skin of a child resents harsh applications. It must be borne in mind that sulphur and mercury, in their local effect, are capable of exciting a catarrhal dermatitis which persists after the scabies has been cured, a circumstance which is signalized by a subsidence of the characteristic itching, and the appearance of an erythema very unlike the original eruption. Eczema may also be engendered by reason of irritating applications. Disinfection of bedding, clothing, and the like, should be carried out by boiling or baking. The underclothing had best not be changed while the treatment is in progress, but the outer garments should be gone over with a hot smoothing-iron. The trousers should be turned wrong side out, and the seams of the legs and pockets carefully ironed. Blankets and comforts may be subjected to the same manipulations. Sherwell's plan of sprinkling powdered sulphur about the bedding is serviceable. It is, of course, best to isolate the patient to minimize the opportunities of spread and simplify the disinfecting process.

It would be desirable, also, for the profession to enjoin upon hotel and boarding-house keepers, Pullman car conductors, and those having charge of public carriers, to use precautions for the protection of the traveling public.

By these simple means, there is no good reason why the progress of the epidemic should not be checked, and many people spared from an affection which is a torture to the flesh and a humiliation to the spirit.

To recapitulate: Scabies exists in the form of an epidemic; it was introduced by returned soldiers from the Spanish-American War; it is on the increase. The recommendations are, that the medical profession, after their attention has been called to the existence of the epidemic, should exert greater diligence in endeavoring to prevent its spread by cultivating more careful methods of diagnosis, and by enjoining upon their patients the necessity for thorough and prompt disinfection.

